# Cryo-EM of a heterogeneous biochemical fraction elucidates multiple protein complexes from a multicellular thermophilic eukaryote

**DOI:** 10.1016/j.yjsbx.2023.100094

**Published:** 2023-08-09

**Authors:** Dmitry A. Semchonok, Fotis L. Kyrilis, Farzad Hamdi, Panagiotis L. Kastritis

**Affiliations:** a Interdisciplinary Research Center HALOmem, Charles Tanford Protein Center, Martin Luther University Halle-Wittenberg, Kurt-Mothes-Straße 3a, Halle/Saale, Germany; b Institute of Biochemistry and Biotechnology, Martin Luther University Halle-Wittenberg, Kurt-Mothes-Straße 3, Halle/Saale, Germany; c Institute of Chemical Biology, National Hellenic Research Foundation, Athens, Greece; d Biozentrum, Martin Luther University Halle-Wittenberg, Weinbergweg 22, Halle/Saale, Germany

**Keywords:** Native cell extracts, Biochemical fraction, Cryo-EM, Single-particle image processing, Simultaneous 3D reconstructions

## Abstract

Biomolecular complexes and their interactions govern cellular structure and function. Understanding their architecture is a prerequisite for dissecting the cell's inner workings, but their higher-order assembly is often transient and challenging for structural analysis. Here, we performed cryo-EM on a single, highly heterogeneous biochemical fraction derived from *Chaetomium thermophilum* cell extracts to visualize the biomolecular content of the multicellular eukaryote. After cryo-EM single-particle image processing, results showed that a simultaneous three-dimensional structural characterization of multiple chemically diverse biomacromolecules is feasible. Namely, the thermophilic, eukaryotic complexes of (a) ATP citrate-lyase, (b) Hsp90, (c) 20S proteasome, (d) Hsp60 and (e) UDP-glucose pyrophosphorylase were characterized. In total, all five complexes have been structurally dissected in a thermophilic eukaryote in a total imaged sample area of 190.64 μm^2^, and two, in particular, 20S proteasome and Hsp60, exhibit side-chain resolution features. The *C. thermophilum* Hsp60 near-atomic model was resolved at 3.46 Å (FSC = 0.143) and shows a hinge-like conformational change of its equatorial domain, highly similar to the one previously shown for its bacterial orthologue, GroEL. This work demonstrates that cryo-EM of cell extracts will greatly accelerate the structural analysis of cellular complexes and provide unprecedented opportunities to annotate architectures of biomolecules in a holistic approach.

## Highlights

• The structural study of protein complexes in crude cell extracts is challenging due to the inherent complexity.
• Partly purified cell extract from a thermophilic fungus retrieves protein complexes.
• Cryo-electron microscopy of cell extract revealed five different protein complexes, at differing resolutions.
• Side-chain features for the 20S proteasome and the Hsp60 were resolved.
• Overall, the results provide the basis for accelerated structural analysis of five different eukaryotic protein complexes in a parallel fashion.

## Graphical abstract

**Figure:**
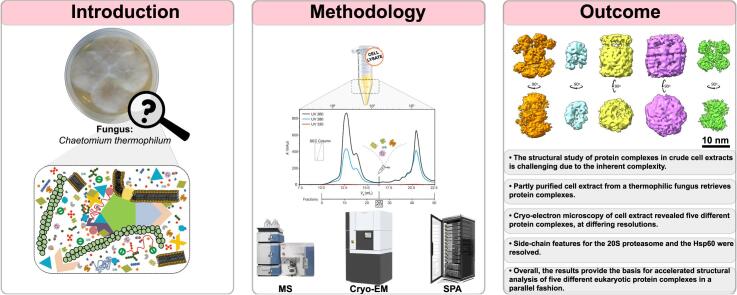

## Introduction

Protein-protein interactions drive most cellular processes, including signalling and metabolism. Cellular pathways often include large biomolecular assemblies composed of multiple copies of one or more polypeptide chains. This is true for nucleocytoplasmic transport (Mackmull et al., 2017, Mosalaganti et al., 2022), mRNA (Cramer, 2019, Osman and Cramer, 2020) and protein synthesis (Schuller and Green, 2018), as well as protein degradation (Förster et al., 2013, Sakata et al., 2021), chaperone activities (Karamanos et al., 2019, Thomas and Tampe, 2019), and pyruvate metabolism (Gray et al., 2014) to name a few. Increased molecular weight is critical because all the processes mentioned above are highly complex and cannot be performed by a single polypeptide chain with a restricted capacity to encode pathway function. To achieve their purpose, large biomolecular assemblies are often regulated by extensive modifications and splicing variants (Kelemen et al., 2013), conformational changes, dynamics and plasticity (Perilla et al., 2015), allosteric communication of distal structural regions (Perica et al., 2012), broad stoichiometric variation (Ori et al., 2016) and transient higher-order assembly (Kastritis and Bonvin, 2013), among many other reported mechanisms (Levy and Onuchic, 2006, Wilson et al., 2018).

Large biomolecular assemblies are notoriously difficult to characterize due to their sheer size and the structural adaptations described above. Ribosome study, as one recent example, from crystallization to structure determination at higher than 3 Å resolution required decades (Selmer et al., 2006, Yonath et al., 1984). However, advances in the field of cryogenic electron microscopy (cryo-EM) (Kuhlbrandt, 2014) have allowed unprecedented insights into large biomolecular assemblies that were previously considered impossible to be structurally analyzed, *e.g.*, the spliceosome (Galej et al., 2016), metabolons (Tuting et al., 2021) and membrane proteins and complexes (Cheng, 2018). Cryo-EM advances in sample preparation, hardware, and software have brought the “resolution revolution” in the field of structural biology, which is currently ongoing (Nakane et al., 2020, Subramaniam, 2019, Yip et al., 2020). Recently, spectacular insights have been provided in the study of complex specimens (Kastritis et al., 2017, von Appen et al., 2015), in addition to their integration with artificial intelligence algorithms (Mosalaganti et al., 2022, Skalidis et al., 2022). Cryo-EM has inherent advantages, namely a) specimen is embedded in vitreous ice and, therefore, closer to the in-solution conformation; b) specimen can be vastly distinct in complexity, spanning from purified proteins to cells. Increasing sample complexity comes with an expense in achievable detail, but recent studies both in single-particle cryo-EM and cryo-electron tomography (cryo-ET) have shown that, for abundant large biomolecular assemblies, higher detail is feasible (Mosalaganti et al., 2022, Skalidis et al., 2022).

A field that has recently emerged with great potential is cryo-EM's structural study of cell extracts (Kastritis et al., 2017). Cell extracts can retain principles of cellular organization (Kyrilis et al., 2019) and are accessible to proteomics, computational and cryo-EM characterization, offering opportunities for identification and analysis of biomolecular assemblies that cannot be identified in cells due to crowding, size and low abundance (Kyrilis et al., 2021a). The sub-nm resolution was achieved for fatty acid synthase (FAS) structure derived from a complex mixture of biomolecular assemblies (Kastritis et al., 2017). Subsequently, other works studied soluble and membrane biomolecular assemblies in bacteria (Su et al., 2021) and protozoa (Ho et al., 2020). These organisms have relatively smaller proteomes than other eukaryotes (Apic et al., 2001), *e.g.*, fungi, the biomolecular assemblies of which may better resemble their human counterparts. However, few studies have been performed that far on native cell extracts of eukaryotes: These include very high molecular weight complexes (>5 megadaltons (MDa)) derived from the thermophilic fungus *Chaetomium thermophilum* (Kastritis et al., 2017, Kyrilis et al., 2021b, Skalidis et al., 2022, Tuting et al., 2021).

In this work, we reproducibly fractionate *C. thermophilum* cell extracts to retrieve biomolecular complexes from a structurally uncharacterized biochemical fraction. This single biochemical fraction comprises ∼ 1 megadalton range complexes, such as the proteasome and the Hsp60. We annotate the proteomic content with mass spectrometry and perform cryo-EM to quantify and visualize the increased specimen complexity. This approach allowed us to eventually resolve the previously unknown architectures of large, biomolecular assemblies in the sub-nm resolution range from a multicellular, thermophilic eukaryote.

## Material and methods

### Sample preparation for electron microscopy

The organism was grown as previously described, as well as the complete biochemical protocol to retrieve the SEC fractions (Kyrilis et al., 2021b). 8 g of *C. thermophilum* mycelium was harvested and either directly used for further purification or stored at −80 °C until usage. Mycelium was lysed in 20 mL Lysis buffer (100 mM HEPES pH 7.4, 95 mM NaCl, 5 mM KCl, 1 mM MgCl2, 0.5 mM EDTA, 5% Glycerol, 1 mM DTT, 10 mg^.^mL^−1^ DNase, Pefabloc 2.5 mM, E-64 40 mM, Bestatin 130 mM, Aprotinin 0.5 mM, Leupeptin 1 mM, Pepstatin A 60 mM) by bead-beating using a FastPrep-24™ 5G. Lysis was carried out in three rounds of 30 s beading time at 4 °C, while the sample rested on ice for 3 min after every cycle. Lysed cells were spun down using at 4,000 *g* for 5 min in order for the large debris, aggregates and non-lysed cells to be removed. The supernatant was centrifuged for 45 min at 100,000*g* and subsequently filtered through a 0.22 μm filter and concentrated by spin filtration at a cut-off of 100 kDa. 500 μl (approx. 30 mg^.^mL^−1^) were injected onto a pre-equilibrated (with Lysis buffer) SEC S4000 Phenomenex HPLC column using an Åkta FPLC system. Flow rate was set to 0.25 mL/min and fraction volume at 250 μl. Bradford measurement was performed to determine concentrations before loading the SEC column as well as the fraction of interest. 3 μl aliquot of the sample eluting in fraction 25, which was chosen due to the specific molecular weight of the complexes included, with a total protein concentration of 0.3 mg^.^mL^−1^ (Bradford measurements) was applied to the glow-discharged (PELCO easyGlow, 15 mA, 25 s) Quantifoil holey carbon-supported grids (R 2/1, 200 mesh). The sample excess was blotted (Whatman #1, blot force 2, blotting time of 6 sec) and plunge-frozen in liquid ethane using an FEI Vitrobot Mark IV at 4 °C and 95 % humidity.

### Electron microscopy data acquisition

Image acquisition was performed on the Thermo Fisher Scientific Glacios equipped with a field emission gun and operated at 200 kV in bright field imaging mode. Movies were recorded using a Thermo Scientific Falcon 3EC Direct electron Detector in counting mode at a nominal magnification of 150,000, corresponding to a pixel size of 0.96 Å^.^pix^-1^ with 30 frames at a total dose of 28 e^-.^Å^−2^ and an exposure time of 30 s per movie.

### Image processing

The dataset of the biochemical fraction derived from SEC consisted of 1109 movies. The raw movies were imported into cryoSPARC v3.2 (Punjani et al., 2017) for the following processing steps. Patch motion correction (Rubinstein and Brubaker, 2015, Zheng et al., 2017) and patch CTF estimation - that is based on a CTF model also implemented in CTFFIND4 (Rohou and Grigorieff, 2015) - was performed.

In detail, the picking of the particles was initially manually performed within cryoSPARC to understand the diversity of particle diameters. Particle diameters were estimated to be ∼ 20 nm and blob picking (Voss et al., 2009) was applied with a 25 nm diameter in cryoSPARC by default.

A total of 287,314 particles were extracted by template-matching particle picking (Sigworth, 2004). After multiple rounds of 2D classification, the main protein complexes with apparent features were sorted. The particles for the defined 2D classes were formed into 5 major groups. For each group, the *ab initio* protocol from cryoSPARC v3.2 was run. As a result, 5 initial 3D maps were obtained. The following 3D refinements with correspondent 3D maps obtained previously were iteratively performed (Table S1). After non-uniform 3D refinement (Punjani et al., 2020), the resulted 3D maps were further analysed and compared with mesophilic counterparts utilising the Scipion software (de la Rosa-Trevin et al., 2016). For identifying each map, a visual inspection of the 3D map, including Omokage shape similarity search of macromolecules (https://pdbj.org/emnavi/omo-search.php) and correlation to the abundance data from mass spectrometry was performed to cross-validate the identified structural signature. Local resolution estimation was performed for all five protein complexes in cryoSPARC v3.2 (Punjani et al., 2017) which has implemented an approach similar to monores (Vilas et al., 2018). Cryo-EM-related parameters for each 3D reconstruction are reported in Table S1. Comparisons to deposited cryo-EM maps and coordinate files were performed using functions implemented in UCSF Chimera (Pettersen et al., 2004), and ChimeraX (Pettersen et al., 2021).

### Model building

The obtained cryo-EM 3D local refinement map of the chaperone Hsp60 resolved at 3.46 Å (FSC = 0.143) was used for model building in Coot (Emsley and Cowtan, 2004). The initial 3D model of UniProt sequence: G0RYB3 was provided by the AlphaFold structure prediction website (https://alphafold.ebi.ac.uk/), produced using AlphaFold Monomer v2.0 (Jumper et al., 2021). The map’s resolution was sufficient to assign protein subunits unambiguously. However, in certain areas, density is low resolved or due to high flexibility at relatively low resolution. The extended flexible parts from each side of the AlphaFold initial model were truncated, representing no density in the experimental cryo-EM 3D map. Rounds of real-space refinement were performed in Phenix (Liebschner et al., 2019) and included simulated annealing protocol without applying symmetry as the refinement is performed in a symmetry-expanded map. Coordinates were manually edited in Coot after each refinement cycle and subjected to further rounds of refinement. The final validation check was performed with MolProbity (Chen et al., 2010) and Phenix validation tools. DALI search (Holm, 2022) was finally performed against the Protein Data Bank to identify structural homologues for Hsp60 models produced by Alphafold and after refinement in the cryo-EM map.

## Results

**Fig. 1:**
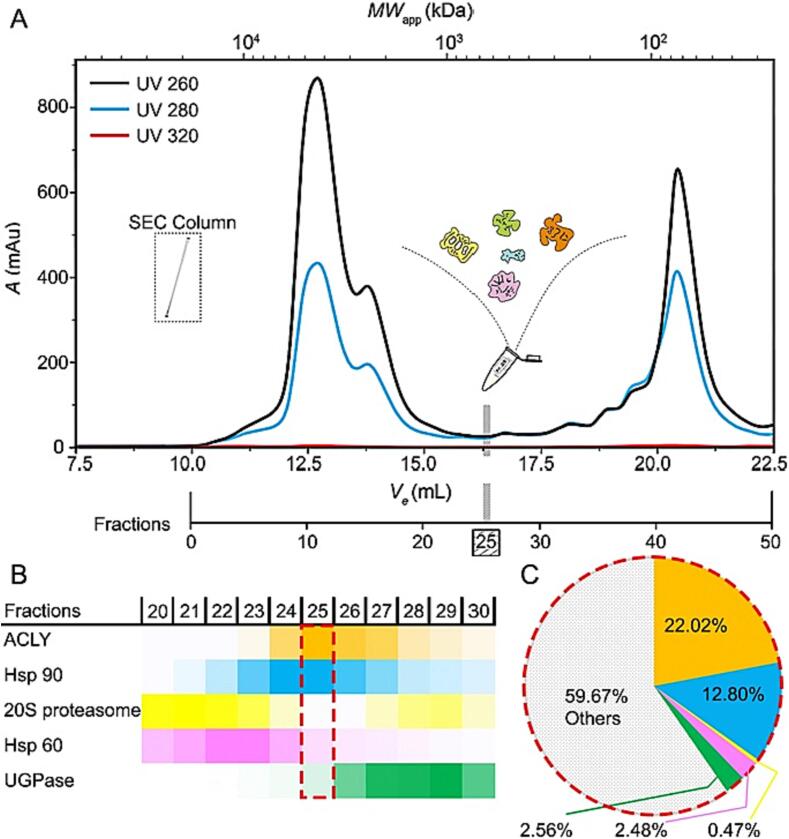
Fractionation of *Chaetomium thermophilum* cell extract and identification of five primary protein complexes in a retrieved biochemical fraction. In (A), *Chaetomium thermophilum* extract is fractionated, and the derived protein complexes are separated based on size. Absorbance units (mAu) are shown monitoring 260, 280 and 320 nm wavelengths. The cartoon drawings within the SEC profile illustrates the five major structural signatures that are distinguishable and are reported in this work. Insert labelled as “SEC column” represents the S4000 Phenomenex HPLC column utilized. (B-C) Analysis of mass spectrometry (MS) data revealed the presence of proteins assembling the studied complexes in the fraction, at variable abundance (color-coding represents iBAQ values while shadowing their relative difference in abundance). (C) Statistics on the relative abundance of protein complexes within the biochemical fraction. For detailed information on the identity of the “Others,” please refer to the published Supplementary Information by *Kastritis et al.* (Kastritis et al., 2017).

Growth and lysis conditions allowed the retrieval of a size-exclusion chromatography (SEC) profile of sufficient resolution and comparable to its previously reported counterpart (Kyrilis et al., 2021b), where multiple peaks across the fractions are discernible, especially in the longer retention times (Fig. 1A). The choice of the organism was also a result of the well-established higher stability of thermophilic complexes in combination with its eukaryotic nature (van Noort et al., 2013). For the selected biochemical fraction (corresponding to ∼ 1 MDa complexes according to standard elution) proteomic search was performed to identify in-fraction protein components. Overall, five protein complexes were identified in this fraction with varying abundance, pointing to a highly heterogeneous biochemical fraction. The fraction includes various protein components of *C. thermophilum* that participate in citrate conversion, protein degradation, chaperone activity and heat shock (Fig. 1B,C), and other metabolic pathways annotated via gene ontology terms and previously described (Kastritis et al., 2017).

### Cryo-EM of a single biochemical fraction visualizes structural signatures of MDa size

**Fig. 2:**
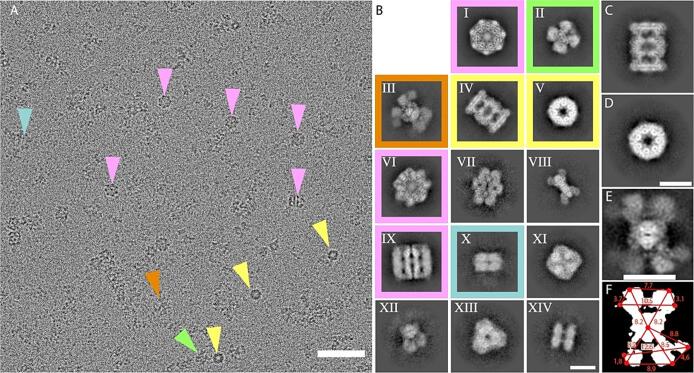
Cryo-EM Analysis of Protein Complexes in a Biochemical Fraction derived from *C. thermophilum* extract. (A) Representative micrograph illustrating the distribution of structural signatures. Colored triangles indicate representatives of the five major protein complexes. Scale bar: 50 nm. (B) Two-dimensional (2D) class averages displaying structural signatures of the five most abundant protein complexes (I-XIV): Hsp60 (I, VI, IX), UGPase (II), ACLY (III), 20S proteasome (IV, V), and Hsp90 (X). Additionally, 2D class averages (VII, VIII, XI, XII, XIII, XIV) correspond to unidentified protein complexes. Scale bar: 10 nm. (C) Side view of the 2D class average representing the 20S proteasome complex, while (D) shows a top view. Scale bar: 10 nm. (E) The 2D class average of the structural signature for ACLY reveals higher intensity values in the core region of the complex, with less defined peripheral subunits due to their variability. (F) A mask derived from the 2D class average projection of ACLY (from E) provides distance measurements in Å, localizing the various subunits relative to the ACLY core.

Initial cryo-EM experiments showed highly concentrated specimen and increased ice thickness, but control of protein concentration via Bradford assays as well as vitrification conditions to optimize the blotting time (8 s), yielding micrographs of superior contrast (Fig. 2A)**.** In these micrographs, various structural signatures can be observed. However, due to the lower molecular weight of the imaged biomolecules (*e.g.*, compared to micrographs depicting larger protein assemblies (Kyrilis et al., 2021b)), visual inspection and subsequent annotation were not feasible. Therefore, a cryo-EM dataset of 1109 movies was collected on Glacios 200 kV cryo-transmission electron microscope, equipped with a Falcon 3EC direct electron detector at a pixel size of 0.96 Å^.^pix^-1^ (Table S1). Overall, an area of 190.64 μm^2^ was imaged, revealing a variety of heterogeneous single particles (Fig. 2A).

### Identification of distinct structural signatures within the biochemical fraction

Following iterative particle picking and 2D classifications, multiple 2D projections showcasing various structural signatures were identified (Fig. 2A, B). The five most abundant complexes, each with their respective 2D class averages, were boxed and represented by specific colors. Notably, signatures IV and V, enclosed within a yellow square in Fig. 2B, were immediately recognized as the side and top views of the 20S proteasome (Fig. 2C, D) (Baumeister et al., 1988). This shape was cross-validated with previous crystallographic (Groll et al., 1997) and cryo-EM data (Danev and Baumeister, 2016). Mass spectrometry (MS) data confirmed the presence of proteins comprising the 20S proteasome in the fraction, although at a low abundance (Fig. 1B, C)**.** Additionally, another discernible structural signature (Fig. 2B, signatures I, VI and IX) resembled the mitochondrial chaperonin Hsp60 (Cheng, 2018, Enriquez et al., 2017), as well as its bacterial counterpart, GroEL (Joseph et al., 2016). Hsp60 was also identified through mass spectrometry, but once again, its abundance in the fraction was low (Fig. 1B). Interestingly, an additional intricate structural signature was observed, consisting of a central structure accompanied by peripheral subunits positioned at an equal distance from the core. This distinct arrangement is highlighted within a boxed area, indicated by a yellowish-orange hue (Fig. 2B-III). Upon closer examination, this specific structural signature exhibited a striking resemblance to the recently determined, full-length structures of ATP citrate lyase (ACLY) and its derived class averages, which were overexpressed (Verschueren et al., 2019, Wei et al., 2020). ACLY was relatively abundant and displayed significant conformational flexibility and overall variation in its external subunits (Fig. 2E, F). All the aforementioned complexes are expected to elute within this molecular weight range, considering their experimentally determined sizes from other eukaryotes and their mesophilic counterparts.

**Fig. 3:**
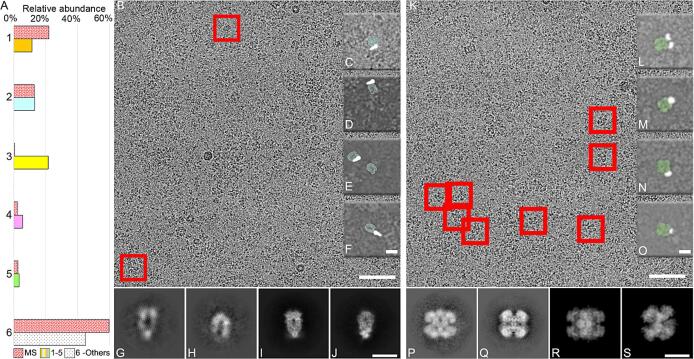
Mass spectrometry and particle abundance of 5 structural signatures in the EM micrographs, and electron microscopy data for the Hsp90 and UGPase protein complexes. (A) Comparison of the relative abundance of five protein complexes identified by mass spectrometry (MS) and single-particle image analysis. The red-dotted pattern bar represents the MS results for the five abundant complexes and the “others” category (number 6). The complexes are labeled as follows: 1 - ACLY (orange), 2 - Hsp90 (light blue), 3 − 20S proteasome (yellow), 4 - Hsp60 (magenta), 5 - UGPase (bright green), and 6 - others (black-dotted). Fraction 3 exhibits the largest disparity between MS and single-particle image analysis, particularly regarding the 20S proteasome complex. This discrepancy could be attributed to disruption during cryo-EM sample preparation or attachment to the carbon support of the holey EM grid. (B) Cryo-EM micrograph displaying higher-order assemblies of Hsp90, with representative examples highlighted by red rectangles. Scale bar: 50 nm. (C-F) Cropped images of Hsp90 higher-order assemblies (Hsp90 with binder) observed in different micrographs. Hsp90 is depicted in light blue, while the binder is shown in white. The crops are low-pass filtered for improved visibility. Scale bar: 10 nm. (G-J) Two-dimensional (2D) class average projections of Hsp90, including (I-J), which are derived from the mesophilic counterparts (EMDB-23214). Scale bar: 10 nm. (K) Cryo-EM micrograph capturing higher-order assemblies of UGPase, with representative examples enclosed in red rectangles. Scale bar: 50 nm. (L-O) Cropped images of UGPase higher-order assemblies (UGPase with binder) observed in different micrographs. UGPase is depicted in green, while the binder is shown in white. The crops are low-pass filtered for improved visibility. Scale bar: 10 nm. (P-S) Two-dimensional class average projections of UGPase, with (R-S) representing projections based on the mesophilic counterparts using PDB ID − 2I5K as a template. Scale bar: 10 nm. (For interpretation of the references to color in this figure legend, the reader is referred to the web version of this article.)

Complexes that were found to be highly abundant and plentiful as single particles within the imaged fraction (Fig. 3A) include Hsp90 and UDP-glucose pyrophosphorylase (UGPase). *In vitro* structures of these complexes in lower molecular weight ranges of approximately 200 kDa (Lee et al., 2021) and 500 kDa (Roeben et al., 2006). Hence, their elution in this fraction cannot be solely explained by their individual molecular weights, unless they are involved in higher-order species. By mapping and class-averaging single particles for each complex from the original micrographs (Fig. 3B, K), we observed their presence within the extract alongside other electron-dense material in close proximity (Fig. 3C-F, 3L-O). The retrieved structural signatures of both complexes exhibit clear architectures resembling the 2D projections observed in their mesophilic counterparts (Fig. 3G-J, 3P-S). Prior studies utilizing mass spectrometry and cross-linking data have revealed the presence of both complexes within native cell extracts, where they participate in higher-order assemblies (Kastritis et al., 2017). However, in the extract, after cryo-EM analysis, they are often captured in their unbound state (Fig. 3G-H, 3P-Q). This could be attributed to the high heterogeneity and plasticity of potential binders, resulting in core scaffolds of potentially heterogeneous and low-contrast higher-order assemblies (Fig. 3B-S).

### *De novo* 3D reconstructions of distinct structural signatures retrieved in the fractions

**Fig. 4:**
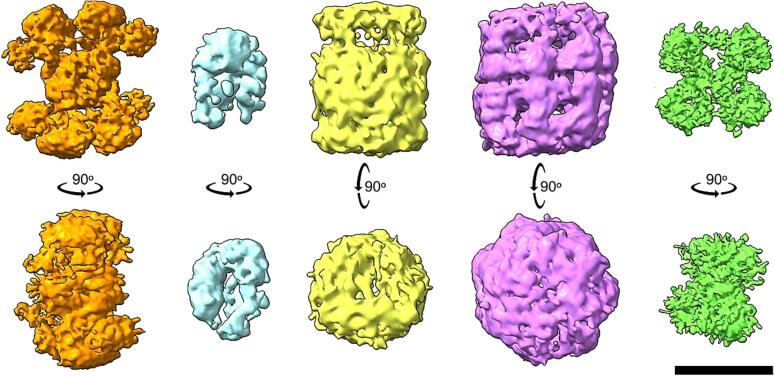
*ab initio* 3D cryo-EM maps of five abundant protein complexes identified within the biochemical fraction. The initial cryo-EM 3D maps of these complexes are presented from left to right: ACLY (orange), Hsp90 (light blue), 20S proteasome (yellow), Hsp60 (magenta), and UGPase (bright green). The row below showcases 90-degree rotated initial models of the complexes. The scale bar represents 10 nm. All cryo-EM 3D maps were generated using the *ab initio* reconstruction protocol from the cryoSPARC 3.1 image processing software. (For interpretation of the references to color in this figure legend, the reader is referred to the web version of this article.)

In order to gain insights into the three-dimensional (3D) architecture of the molecules represented by the previously described structural signatures, *de novo* reconstructions were performed for each distinct signature. The respective number of particles used for de novo reconstruction were as follows: ATP citrate lyase – 32,549 particles, Hsp90 – 25,713 particles, 20S proteasome – 4,231 particles, Hsp60 – 10,578 particles, and UGPase – 9,678 particles (Fig. 4). While the particle numbers obtained through label-free mass spectrometry (MS) quantification did not quantitatively match their relative abundance within the fraction (Fig. 3A), the qualitative results were consistent. The resulting 3D maps exhibited similarities to their mesophilic counterparts and shared global structural features with mesophilic atomic models and 3D maps (Fig. S1A-D) (Fernandez-Gimenez et al., 2021).

### Retrieved maps, after refinement, elucidate stoichiometries, symmetry information, and additional binding partners

An overview of methodological and statistical details for all reconstructed maps is shown in Table S1. Briefly, the abundance of collected datasets within the studied extract doesn’t allow resolving the UGPase complex at high resolution: more particle projections of different views are required to complete the 3D map (Euler sphere). That is why only the initial 3D map recontraction of this complex is available (Fig. 4 – bright green) (Fig. S2A,B). Of particular interest, however, is a consistent 3D reconstruction determined for the ATP-citrate lyase at 7.92 Å (FSC = 0.143) and 7.01 Å (FSC = 0.143) with C1 and D2 symmetry applied correspondently (Fig. S2C,D), which directly shows the localization of the corresponding subunits and the inter-subunit distances within the reconstruction (Fig. S2E). The outer coenzyme A (CoA) binding subunits exhibit the highest flexibility, as reflected by the local resolution estimation of the derived map (Fig. S2F). Another interesting asymmetric reconstruction is Hsp90, which resolved at 10.19 Å (FSC = 0.143) (Fig. S2G-I; Table S1)**.** Hsp90 is captured in a closed state (Fig. S1B; Fig. S2H), and all domains (N-ter, MD and C-ter) and their separation are observed, resembling the Hsp90 cryo-EM map (EMD-23214) from the *H. sapiens* orthologue (Lee et al., 2021) **(**Fig. S1B**)**.

**Fig. 5:**
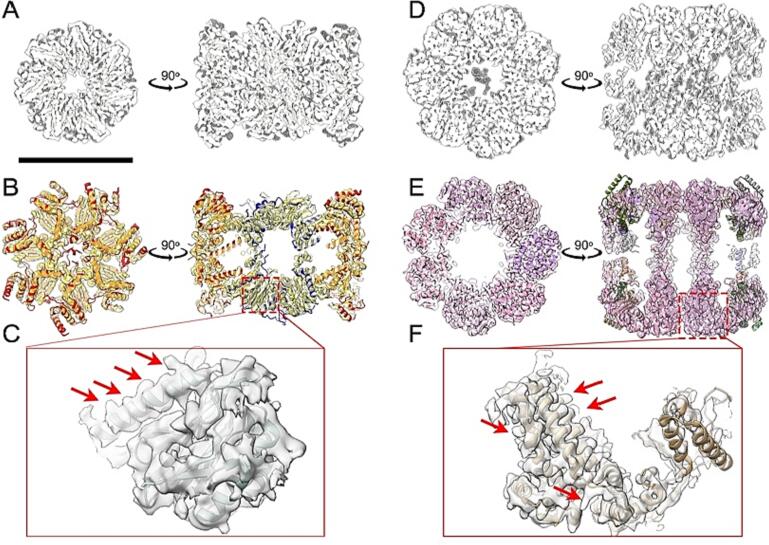
Structural characterization of 20S proteasome and Hsp60 from *C. thermophilum*. (A) Aligned slices of 3D maps of the 20S proteasome without symmetry (C1) and with D7 symmetry are superimposed. The shaded area represents the C1 scaffold, while the white area represents the D7 symmetry. Differences in the C1 scaffold between the two 3D maps can be observed. (B) Sliced 3D map of the 20S proteasome (top and side view) after symmetry expansion and local refinement using cryoSPARC 3.1. The map is fitted with the mesophilic counterpart (PDB: 7LS5). The α subunit is marked in red, and the β subunit is marked in blue. A good fit of the secondary structure to the atomic model (PDB: 7LS5) can be observed in the cryo-EM 3D map. (C) Zoomed-in view of the cryo-EM 3D map of the 20S proteasome with the fitted model of the mesophilic counterpart (PDB: 7LS5). In this area, the β-type subunit of chain V contains catalytically active threonine residues at its N terminus. The density for side chains in the cryo-EM 3D map is indicated with red arrows. (D) Aligned slices of 3D maps of Hsp60 with C1 symmetry and D7 symmetry are superimposed and differences in the C1 scaffold 3D map can be observed. (E) Sliced local refined 3D map of Hsp60 (top and side view) with the fitted mesophilic counterpart (PDB: 7AZP). The fitted chains are differently coloured, highlighting higher coverage of the central part of Hsp60 (in pink). The cryo-EM 3D map of Hsp60 recapitulates secondary structure elements after rigidly fitting its mesophilic counterpart (PDB: 7AZP). (F) Zoomed-in view of the cryo-EM 3D map of Hsp60 (chain D, 1–525 residues, PDB: 7AZP). The side-chain densities derived in the cryo-EM 3D map is indicated with red arrows. The scale bar for panels A, B, D, and E is 10 nm. (For interpretation of the references to color in this figure legend, the reader is referred to the web version of this article.)

The rest of the reconstructed maps, namely the eukaryotic thermophilic 20S proteasome and mitochondrial Hsp60, reached higher resolution (Fig. S3A-H). The 20S proteasome reached 3.97 Å (FSC = 0.143) while the mitochondrial Hsp60 reached 3.65 Å (FSC = 0.143) resolution, respectively (Fig. S3B,G). After symmetry expansion, both maps further improved (Fig. S3C,H). Due to the higher rotational symmetry implementation that governs both complexes (D7), the signal-to-noise ratio boosts the present molecular details. No symmetry (C1) application for both complexes reached lower resolutions (Fig. S3A,F), but the secondary structure is visible and additional molecular details are observed, such as densities inside complex scaffolds, especially for Hsp60 (Fig. 5C,F). Hsp60 is captured without Hsp10, although Hsp10 was present in the quantified mass spectrometry data (Kastritis et al., 2017). This is also in line with the fact that the cryo-EM map of Hsp60 did not show the characteristic symmetrical “American football”-shaped complex (Klebl et al., 2021).

Another significant image processing aspect is the derived molecular details after applying the symmetry expansion (Punjani et al., 2017), where both complexes are resolved with the highest resolution in their core components (Fig. 5B,E), (Fig. S3C,H). Local resolution calculations show that side-chain resolution is visible and captured, for example, in proteasomal subunits α and β (Fig. 5B). Similar high-resolution insights are also derived for the Hsp60, where side-chain resolution is visible for its multimeric state at its core (Fig. 5E,F). The observed local resolution distribution for both complexes also agrees with previous observations regarding (a) the stability of 20S proteasome, its assembly from polymerizing β rings, interacting with the less stable α rings (Zwickl et al., 1999) and (b) the highly stable homomultimeric interaction interfaces of Hsp60 and the increasing flexibility of the Hsp60 structure towards the outer surface, which serves as a scaffold for other protein–protein interactions, including docking of Hsp10.

### The endogenous Hsp60 exhibits abundant rigid body-displacement of intermediate and apical domains

Resolution for the Hsp60 cryo-EM map after symmetry expansion (3.46 Å, FSC = 0.143) was reasonable to derive a molecular model satisfying the cryo-EM data. We started by downloading the Alphafold model corresponding to the identified *C. thermophilum* sequence (Uniprot ID: G0RYB3). The model (Fig. S4A) had high pLDDT and PAE scores (Fig. S4A,B). After cycles of refinements with COOT and PHENIX (see Materials and Methods), the final model was fitted in the cryo-EM map and was of acceptable quality (Table S2) and fit (Fig. S4C).

**Fig. 6:**
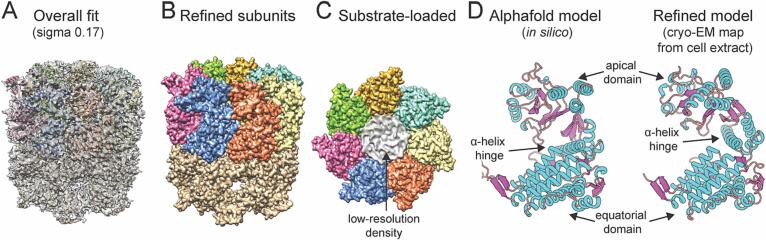
Molecular insights into the *C. thermophilum* Hsp60 retrieved from native cell extracts. (A) fitting of complete Hsp60 is covered at low density threshold due to the uneven resolution distribution of the cryo-EM map (Fig. S3E). (B) Refined *C. thermophilum* Hsp60 monomers in the cryo-EM map exhibits the typical 7-fold rotational symmetric barrel architecture. (C) Low-resolution density is captured in the entrance of the central channel of Hsp60, indicating possible cellular binders. (D) Structure-based refinement of the predicted Alphafold model for *C. thermophilum* Hsp60 suggests that the endogenous state of the molecule is mediated by a hinge-like conformational change in an α-helical element located at the intermediate domain.

Overall, at relatively low map threshold we observed that (a) flexible N- and C- termini are not recapitulated, and, therefore, were truncated from the Alphafold model; (b) the map resolution was heterogeneous, reaching higher values at the Hsp60 equatorial domain, located mostly around the nucleotide binding site, while the apical domain was resolved at lower resolution (Fig. S3E). After refinement of the Alphafold model in the map (Fig. 6A-B), a low-resolution density appeared within the central hole of the Hsp60 (Fig. 6C).

The Alphafold model predicts a substantially different conformation of Hsp60 when compared to the experimentally-refined model in the endogenous map **(**Fig. 6D**)** which can be attributed to the possible binding of substrates, thus acquiring a substrate-bound conformation (Fig. 6D). The refined experimental model exhibits a hinge movement that significantly displaces the apical domain relative to the equatorial domain of Hsp60. It is quite intriguing that relatively lower pLDDT scores corresponded to this exact hinge region (Fig. S4A, light blue). A DALI search of both pre- and post- refined structures yielded similarities to distinct GroEL prokaryotic states (GroEL is evolutionary related to Hsp60): The Alphafold model resembled mostly the GroEL–ADP in the relaxed (R) state (PDB-ID: 4KI8 (Fei et al., 2013), 2.3 Å root-mean-square deviation, Cα-RMSD) while the refined structure shared higher similarity to the GroEL–ATPγS crystal structure (PDB-ID: 1SX3 (Chaudhry et al., 2004), 2.1 Å Cα-RMSD). Considering the similarities above, it is concluded that the Hsp60 found in native cell extracts is, on average, in a state where both the intermediate (hinge) and apical domains are displaced as a single rigid body when compared with unliganded Hsp60 (structure predicted by Alphafold), a stable state identified also for GroEL *in vitro* (Bukau and Horwich, 1998).

## Discussion

### Structural analysis of native cell extracts to complement cryo-electron tomography of mixtures and cells

Cryo-ET and subtomogram averaging have recently provided spectacular insights into the higher resolution analysis of vitrified cells (Bauerlein and Baumeister, 2021), for example, studying the nuclear pore complex (Allegretti et al., 2020), the ribosome (O’Reilly et al., 2020, Xue et al., 2022) and the proteasome (Sakata et al., 2021), as well as other biomolecular complexes; a common denominator for all those is their relatively high abundance in-cell, a reasonable consideration for any cryo-ET sample (Ng and Gan, 2020). Indeed, the closest-to-native imaging that can be achieved for protein complexes and their interactions is within intact cells. Recent advances in cryo-ET image processing allow comparable resolutions to those of single-particle analysis: A recent, similar work to ours has shown that utilizing cryo-ET, *T. acidophilum* proteasomes -in a mixture with *E. coli* ribosomes- can reach ∼ 4.7 Å resolution (Khavnekar et al., 2023). However, increasingly less abundant protein complexes are difficult to capture within the highly complex environment of a cell. Therefore, enrichment methods of protein complexes by gentle lysis of millions of cells and subsequent coarse separation of extracts can provide further insights into macromolecular native states, something we have performed in this study. The strengths and limitations of structurally characterizing cell extracts and their applicability of retaining native interactions have been previously carefully discussed (Kyrilis et al., 2019) and also applied to this work. One of the major concerns is that cellular lysis greatly reduces protein concentration, and consequently, loss of transient interactions can occur. Cross-linking can retain those interactions, but again, the complexes in the cell extract are chemically fixed. In addition, non-specific interactions can be immediately formed due to lysis-induced molecular proximities. Despite those limitations, visualization of protein complexes in cell extracts indeed provides a bridge for a better understanding of protein complexes studied *in vitro* and *in cellulo*, especially for those with lower cellular abundance because biochemical enrichment is performed.

In this work, a single biochemical fraction from a thermophilic eukaryote was studied by applying high-resolution cryo-electron microscopic methods and subsequent image processing of the imaged specimen. The specific biochemical fraction analyzed herein is of significant complexity, including five protein complexes in a continuum of abundance visualized by cryo-EM and estimated from label-free mass spectrometry data. It is of note that structural analysis of a eukaryotic biochemical fraction has not been reported to date, except from previous reports on much higher molecular weight fractions such as structures of the 2.6 MDa structure of fatty acid synthase (Kastritis et al., 2017) and the 10 MDa metabolon of the pyruvate dehydrogenase complex (PDHc) and higher-order complexes found in such early-eluting biochemical fractions (Kyrilis et al., 2021b, Skalidis et al., 2022, Tuting et al., 2021). Notably, after reporting the feasibility of *C. thermophilum* native cell extracts for structural characterization (Kastritis et al., 2017), spectacular works have studied complex mixtures of biomolecules (Ho et al., 2020, Su et al., 2021) and even derived from Protista using sucrose gradient fractionation (Ho et al., 2020), or like in our studies, size-exclusion chromatography of prokaryotic extracts (Su et al., 2021). The latter work also applied image processing protocols to reconstruct cryo-EM 3D maps of high-resolution membrane proteins (Su et al., 2021). Therefore, in addition to the advances mentioned above in the structural characterization of cell extracts, this work provides the first structural analysis of a highly complex biochemical fraction comprised of ∼ 1 MDa complexes from a multicellular eukaryote.

The hope of using cell extracts for structural analysis with electron microscopy is not new: TEM of purified cell extracts has been performed for more than 50 years (Harris, 1968) and, in most cases, is followed by further isolation of the complex of interest, *e.g.,* for the proteasome (Mao, 2021). Only recent advances, however, have allowed unprecedented developments in the field, such as the development of computational approaches to directly annotate the cryo-EM map, provided it is of a side-chain resolution (Chang et al., 2022, Chojnowski et al., 2022, Ho et al., 2020, Jamali et al., 2022, Skalidis et al., 2022). In particular, Ho et al. have developed such tools in a fractionated cell extract derived from the unicellular eukaryotic parasite *Plasmodium falciparum* for substantially smaller protein complexes (Ho et al., 2020) than those we report herein. Despite that, we believe our work has merit because those five complexes reported here have not been structurally characterized to date in a cellular extract from a multi-cellular eukaryote; their thermophilic origin makes this specimen and the residing complexes attractive for future structure-based studies. However, the cryo-EM maps have to be treated with caution when comparing to mesophilic counterparts, as differences observed could be attributed to distinct image processing protocols or technical differences. For the case of Hsp60, for which we derived an atomic model at ∼ 3.5 Å resolution, such comparison is not feasible, as the state in which Hsp60 was captured in the cell extracts has not been structurally characterized in a mesophilic counterpart.

In addition, future benchmarking of particle-picking strategies, *e.g.*, other pickers, different blob diameters or particle-picking densities, could further reveal more structural signatures in the studied fractions. This can be really impactful if more data is collected for all biochemical fractions that capture eukaryotic complexes from *C. thermophilum*. This study provides various insights into a structural analysis of eukaryotic extracts; It is demonstrated that it is feasible to identify and reconstruct multiple structural signatures. This provided the advantage of extracting valuable information regarding their architecture, protein composition, domain organization, stoichiometry, and symmetry. In addition, highly symmetric complexes, such as the 20S proteasome and the Hsp60, can be reconstructed to resolutions close to the 3.5–3.8 Å regime (FSC = 0.143), with the opportunity to provide unprecedented insights into structure–function studies. Especially after molecular modeling of Hsp60, it is concluded that a conformational change that is also described for its prokaryotic ortholog, GroEL, is accurately captured within native cell extracts. This conformation of Hsp60 that shares high similarity to the GroEL–ATPγS crystal structure (PDB-ID: 1SX3 (Chaudhry et al., 2004), 2.1 Å Cα-RMSD) proposes the utilizing of native cell extracts to annotate potential cellular states of biomacromolecules.

Overall, all aforementioned observations described for the five protein complexes from *C. thermophilum* native cell extracts validate the very high conservation in their higher-order structure, not only between different eukaryotes but also between a thermophile and its mesophilic counterparts. It is also likely that lower molecular weight complexes eluting in a fraction where 1 MDa complexes are expected will further provide information for their participation in higher-order biomolecular interactions and formed complexes. Although this was not a major focus of this study, data for the formation of protein communities have already been presented for other complexes present in biochemical fractions, such as FAS (Kastritis et al., 2017) and PDHc (Tuting et al., 2021) with the latter especially forming protein communities of metabolons which were highly active.

As expected, more acquired micrographs and increased particle numbers will allow more protein complexes to be deciphered and reconstructed from the biochemical fractions, improve achievable resolution, further access less abundant structural signatures, and gain information regarding their composition. These advances can occur probably within the following years with advances in imaging (Bai, 2021, Hamdi et al., 2020) and analysis protocols (Sorzano et al., 2021), which are already being placed for applications in single-particle analysis (Kimanius et al., 2021) and, in the future, cryo-electron tomography of cell extracts, *e.g.,* with workflows integrated into image analysis software (Jimenez de la Morena et al., 2022). Lastly, the inclusion of AlphaFold2.0 (Jumper et al., 2021) and other artificial intelligence tools for structural biology, such as *findmysequence* (Chojnowski et al., 2022), into cryo-EM analysis pipelines for native cell extracts (Skalidis et al., 2022) will additionally boost the interpretation of cryo-EM maps from native sources.

## Conclusions

Imaging cell extracts and retrieving structural signatures from a heterogeneous specimen that could retain aspects of cellular function (Kyrilis et al., 2019, Kyrilis et al., 2021a, Skalidis et al., 2022), shows the strength of cryo-EM assisted by MS characterization of biological specimen. In addition, accessing a higher level of structural organization within cell extracts is feasible, as binders and scaffold molecules can be retrieved. Their unambiguous identification within the cryo-EM data is expected to complement cryo-ET studies. This method is expected in the future to provide proximity and spatial information regarding in-extract protein communities. Finally, future developments in our reported work can not only be considered methodologically (*e.g.,* combination with cryo-ET, computational modelling, other complementary fractionation methods, cross-linking MS) but also in a cellular or biological context. Examples include induction of stress, change of growth conditions, adding chemicals that could be probed, and observing correlated structural adaptations in the biochemical fraction complexome with cryo-EM. It is also expected that given enough material is retrieved after, *e.g.*, purification of specific organelles, organelle-enriched protein complexes will then be further annotated. Overall, despite the known limitations of studying native cell extracts for understanding cellular function, previously discussed for all different steps in their structure characterization (Kyrilis et al., 2019) and computational aspects (Kyrilis et al., 2021a), our reported results indeed provide a basis for the structural analysis of native eukaryotic complexes and annotation of their biomolecular architectures in a systematic, large-scale level.

## CRediT authorship contribution statement

**Dmitry A. Semchonok:** Software, Formal analysis, Data curation, Visualization, Validation, Writing – review & editing. **Fotis L. Kyrilis:** Methodology, Data curation, Visualization, Writing – review & editing. **Farzad Hamdi:** Methodology, Data curation. **Panagiotis L. Kastritis:** Conceptualization, Software, Formal analysis, Investigation, Resources, Data curation, Visualization, Validation, Writing – original draft, Supervision, Project administration, Funding acquisition.

## Declaration of Competing Interest

The authors declare that they have no known competing financial interests or personal relationships that could have appeared to influence the work reported in this paper.

## Data Availability

All data are available through EMPIAR, PDB and EMDB databases
