# Supplementary material for: Cryo-EM of a heterogeneous biochemical fraction elucidates multiple protein complexes from a multicellular thermophilic eukaryote
Source: J Struct Biol X. 2023 Aug 9;8:100094. doi: 10.1016/j.yjsbx.2023.100094 (PMC10451023; doi:10.1016/j.yjsbx.2023.100094)
Supplement: Supplementary data 1 [file mmc1.pdf]

SUPPLEMENTARY MATERIAL

Cryo-EM of a heterogeneous biochemical fraction elucidates multiple protein complexes from a multicellular thermophilic eukaryote.

Dmitry A. Semchonok[a], Fotis L. Kyrilis[a,b,c], Farzad Hamdi[a], Panagiotis L. Kastritis[a,b,c,d]*

[a] *Interdisciplinary Research Center HALOmem, Charles Tanford Protein Center, Martin Luther University Halle-Wittenberg, Kurt-Mothes-Straße 3a, Halle/Saale, Germany.*

[b] *Institute of Biochemistry and Biotechnology, Martin Luther University Halle-Wittenberg, Kurt-Mothes-Straße 3, Halle/Saale, Germany.*

[c] *Institute of Chemical Biology, National Hellenic Research Foundation, Athens, Greece.*

[d] *Biozentrum, Martin Luther University Halle-Wittenberg, Weinbergweg 22, Halle/Saale, Germany.*

Abbreviations: 2D, Two-dimensional; 3D, Three-dimensional; ACLY, ATP citrate lyase; C1, Lowest symmetry group (no symmetry); C2, Two-fold rotational symmetry; *C. thermophilum*, *Chaetomium thermophilum*; CoA, Coenzyme A; cryo-EM, Cryo-electron microscopy; cryo-ET, Cryo-electron tomography; D2, Dihedral symmetry of the 2nd order; D7, Dihedral symmetry of the 7th order; EMDB, Electron microscopy Data Bank; FAS, Fatty acid synthase; FSC, Fourier Shell Correlation; Hsp10, Heat shock protein 10 kilodaltons; Hsp60, Heat shock protein 60 kilodaltons; Hsp90, Heat shock protein 90 kilodaltons; kV, Kilovolt; MDa, Megadalton; MS, Mass Spectrometry; N-ter, MD and C-ter N-terminus, middle domain, and C-terminus; PDB, Protein Data Bank; PDHc, Pyruvate dehydrogenase complex; SEC, Size exclusion chromatography; UGPase, UDP-glucose pyrophosphorylase.

* Corresponding author.

E-mail address: panagiotis.kastritis@bct.uni-halle.de (P. L. Kastritis).

Table S1. Sample preparation, Microscopy, Data processing and Validation.

	(a) ATP citrate lyase	(b) Hsp90	(c) 20S proteasome	(d) Hsp60	(e) UDP–glucose pyrophospho-rylase
**Sample preparation**					
Concentration [mg/ml]	0.3				
Sample volume [µl]	3.5				
Grid type	Quantifoil R 2/1 Cu 200				
Glow discharge time [s]	25				
Glow discharge current [mA]	15				
Glow discharge sample polarity	Negative				
Glow discharge atmosphere	residual air				
Glow discharge pressure (Pa)	40				
Chamber temperature [°C]	4				
Chamber humidity [%]	95				
Blot time [s]	6				
**Microscopy**					
Magnification	150 000				
Voltage (kV)	200				
Focal length (mm)	3.4				
Cs (mm)	2.7				
Objective Aperture (µm)	100				
Number of movies	1109				
Electron exposure (e–/Å^2)	30 frames (1 e-/frame)				
Defocus range (µm)	-0.5 to -1.5				
Pixel size (Å)	0.9612				
Symmetry imposed	C1 // D2	C1	C1 // D7 // Symmetry expanded local refinement	C1 // D7 // Symmetry expanded local refinement	C2
Initial particle images (no.)	287314				

Final particle images (no.)	32669	37712	4433 (62062 for symmetry expanded best 3.74 Å resolution)	15942 (223188 for symmetry expanded best 3.46 Å resolution)	9678
Map resolution (Å)/ B-factor (Å^2)<br><br>FSC threshold	7.92 / 865.6 // 7.01 / 687.9<br><br>0.143	10.19<br><br>0.143	7.42 / -369.4 // 3.97 / -134.0 // 3.74 / -129.5<br><br>0.143	6.74 / -475.8 // 3.65 / -125.5 // 3.46 / -124.6<br><br>0.143	-----
Map resolution range (Å)	6 – 11	9 – 10	3.5 – 6	3.25 – 6.5	–
**Mesophilic counterpart** (emd or PDB code)	emd – 20903	emd – 23214 PDB – 7L7J	emd – 23502 PDB – 7LS5	emd – 3415 PDB – 7AZP	PDB – 2i5k

Table S2. Hsp60 cryo-EM map and model validation statistics

	HSP60 (EMDB-17629) (PDB 8PE8)
**Data collection and processing**	
Magnification	150 000
Voltage (kV)	200
Electron exposure (e–/Å^2)	30 frames (1 e-/frame)
Defocus range (μm)	-0.5 to -1.5
Pixel size (Å)	0.9612
Symmetry imposed	D7
Initial particle images (no.)	287314
Final particle images (no.)	15942
Map resolution (Å)<br>FSC threshold	3.46
Map resolution range (Å)	3.25 – 6.5
**Refinement**	
Initial model used (PDB code)	Alphafold, G0RYB3
Model resolution (Å)<br>FSC threshold	3.46<br>0.143
Model resolution range (Å)	3.4/3.5/4.0
Map sharpening *B* factor (Å^2)	-124.6
Model composition<br>Non-hydrogen atoms<br>Protein residues<br>Ligands<br>Chains	 0<br>7392<br>0<br>14
B factors (Å^2)<br>Protein<br>Ligand	 29.03/290.65/160.92<br>---
R.m.s. deviations<br>Bond lengths (Å)<br>Bond angles (°)	 0.002 (0)<br>0.528 (0)
Validation<br>MolProbity score<br>Clashscore<br>Poor rotamers (%)	 1.81<br>11.86<br>0.48
Ramachandran plot<br>Favored (%)<br>Allowed (%)<br>Disallowed (%)	 96.58<br>3.42<br>0.00
Map-model cross-correlation (model vs data)<br>CC (mask)<br>CC (box)<br>CC (peaks)<br>CC (volume)	 0.77<br>0.71<br>0.62<br>0.76

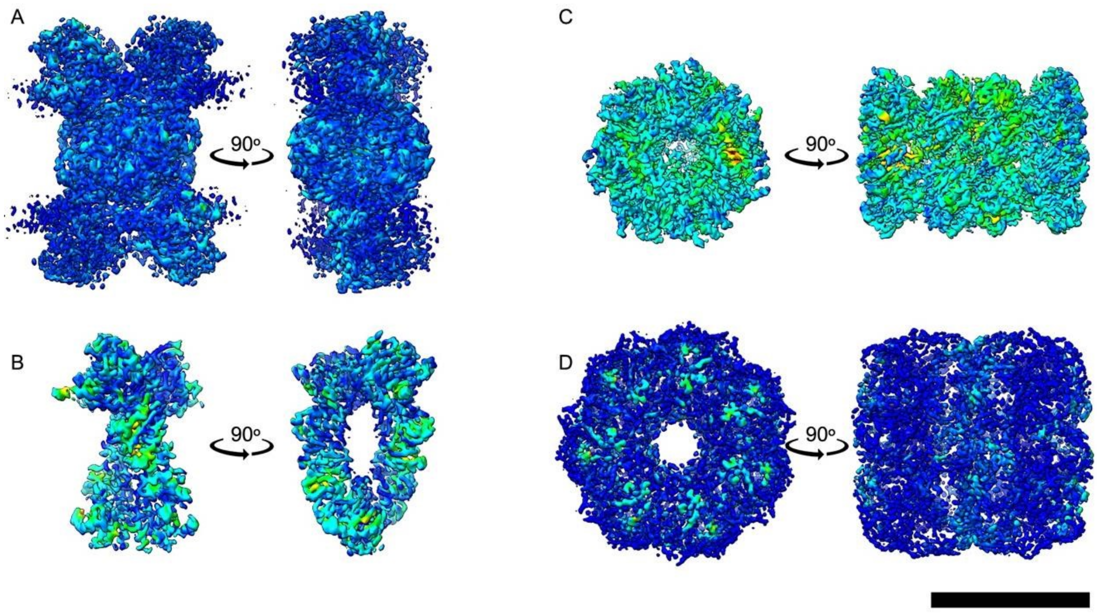

Figure S1. Protein Complexes in *C. thermophilum*: Structural Characteristics and Comparisons of cryo-EM maps. (A) Comparison of cryo-EM three-dimensional (3D) maps between the ACLY protein complex in a biochemical fraction and its counterpart EMD-20903, shown from top and side views. Volume comparison was performed using the tools communicated by Fernández-Giménez et al. (2021). (B) Comparison of cryo-EM 3D maps between the Hsp90 protein complex in a biochemical fraction and its counterpart EMD-23214, shown from top and side views. (C) Comparison of cryo-EM 3D maps between the 20S proteasome complex in a biochemical fraction and its counterpart EMD-23502, shown from top and side views. (D) Comparison of cryo-EM 3D maps between the Hsp60 complex in a biochemical fraction and its counterpart EMD-3415, shown from top and side views. Scale bar: 10 nm. Overall, the four compared maps (except for UGPase) were aligned, overlaid with their mesophilic counterparts (Table S1), and normalized as described by Fernández-Giménez et al. (2021). The cryo-EM maps of the protein complexes are depicted as top and side views, with colors representing the degree of similarity between the input maps, ranging from dark blue (smallest difference) to red (largest difference). Scale bar: 10 nm.

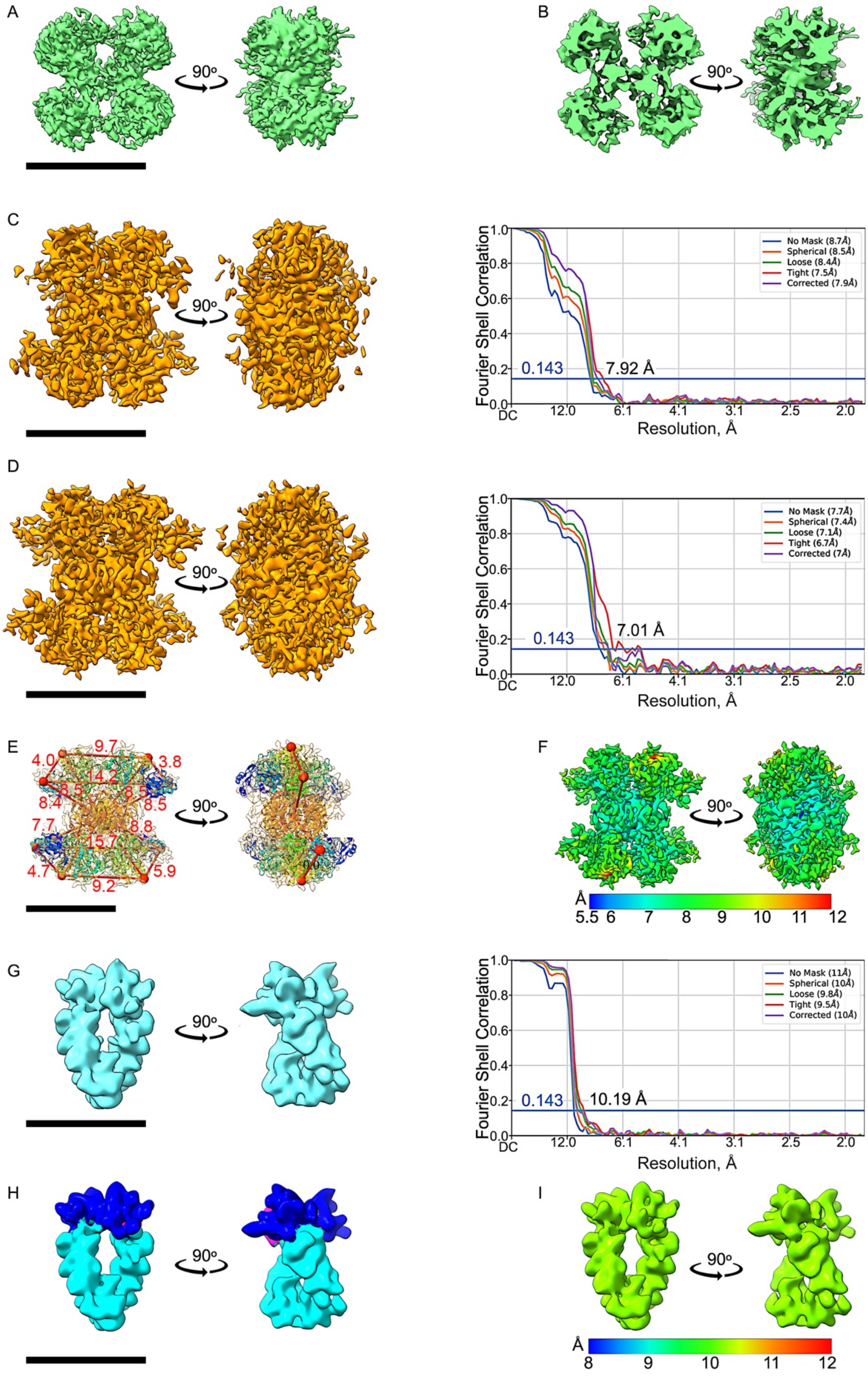

Figure S2. Reconstruction and characterisation of UGPase, ACLY and Hsp90 complexes from *C. thermophilum*. (A) Cryo-EM 3D map reconstruction of UGPase (top and side view). The scale bar is 10 nm. (B) Same as A with a section through the centre of the map. The obtained resolution does not allow the identification of the secondary structure of the UGPase complex. The scale bar is 10 nm. (C) Cryo-EM 3D map reconstruction of ACLY without (C1) imposed rotational symmetry (top and side view). Below are the

correspondent FSC curves. The scale bar is 10 nm. (D) Same as C with D2 rotational symmetry imposed (top and side view). Below are the correspondent FSC curves. The scale bar is 10 nm. (E) Cryo-EM 3D map reconstruction of ACLY with imposed D2 rotational symmetry and fitted in counterpart model PDB: 6UUZ (top and side views). The distances are represented all over the structure, from the sides to the centre. The scale bar is 10 nm. (F) Local resolution of final 3D map of ACLY with D2 symmetry. The scale bar is 10 nm. (G) Cryo-EM 3D map reconstruction of Hsp90 (top and side view). Below are the correspondent FSC curves. The scale bar is 10 nm. (H) Cryo-EM 3D map reconstruction of Hsp90 with fitted in counterpart PDB: 7L7J. The N-terminal domain with ATP-binding domains is coloured in blue. The pink-coloured area represents the possible location of Prostaglandin E synthase 3 (from PDB: 7L7J). The scale bar is 10 nm. (I) Local resolution of final 3D map of Hsp90. The uniform resolution is observed for the Hsp90 closed complex.

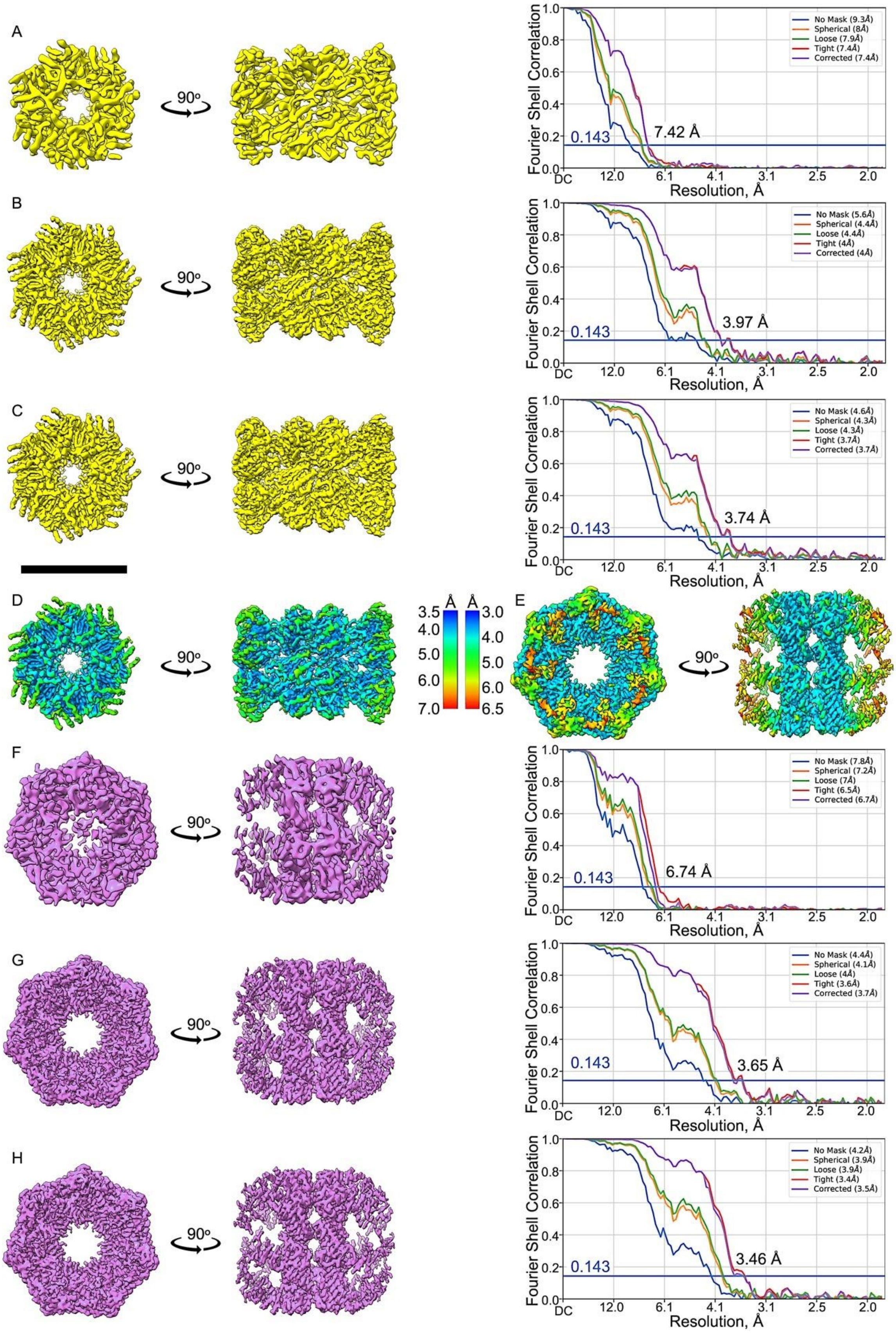

Figure S3. Reconstruction and characterisation of 20S proteasome and Hsp60 complexes from *C. thermophilum*. (A) Cryo-EM 3D map reconstruction of 20S proteasome (top and side view) without rotational symmetry applied (C1). Opposite – the FSC curves of the final density map (FSC = 0.143) with 7.42 Å final resolution. (B) Cryo-EM 3D map reconstruction of 20S proteasome (top and side view) with D7 rotational symmetry applied. Opposite – the FSC curves of the final density map (FSC = 0.143) with 3.97 Å final resolution. (C) Cryo-EM 3D map reconstruction of 20S proteasome (top and side view) with D7 symmetry

expansion refinement. Opposite – the FSC curves of the final density map (FSC = 0.143) with 3.74 Å final resolution. (D) Local resolution 3D map of 20S proteasome (top and side view). Small resolution variations can be observed. (E) Local resolution 3D map of Hsp60 (top and side view). A bigger variation is observed on the edges of the cryo-EM map, where the Column density is faint. (F) Cryo-EM 3D map reconstruction of Hsp60 (top and side view) with C1 rotational symmetry applied. Opposite – the FSC curves of the final density map (FSC = 0.143) with 6.74 Å final resolution. (G) Cryo-EM 3D map reconstruction of Hsp60 (top and side view) with D7 rotational symmetry applied. Opposite – the FSC curves of the final density map (FSC = 0.143) with 3.65 Å final resolution. (H) Cryo-EM 3D map reconstruction of Hsp60 (top and side view) with D7 symmetry expansion refinement. Opposite – the FSC curves of the final density map (FSC = 0.143) with 3.46 Å final resolution. The scale bar for all 3D cryo-EM reconstructions is 10 nm.

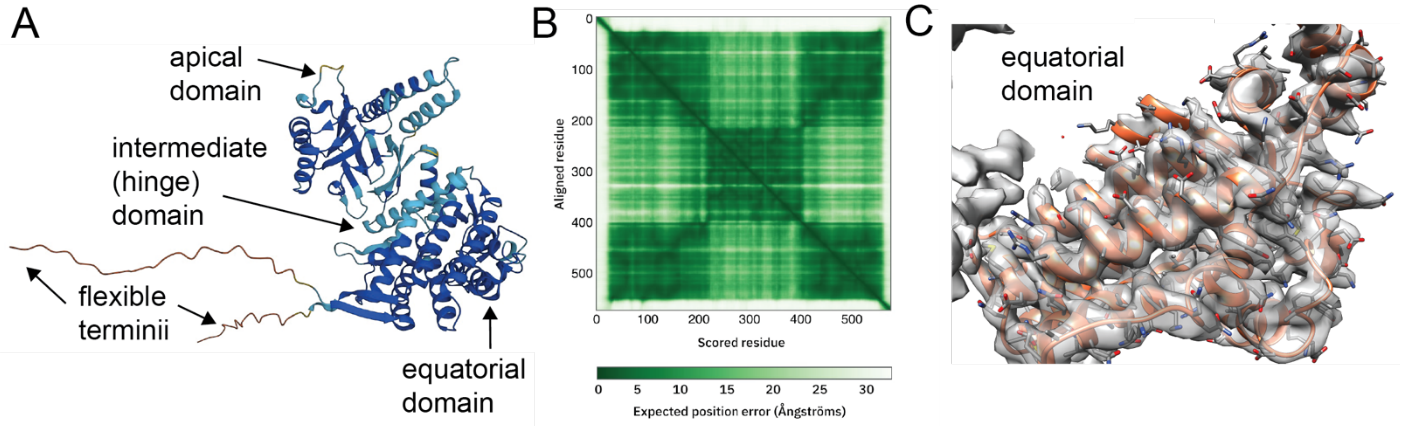

Figure S4. Structural analysis of C. thermophilum Hsp60. (A) Alphafold model downloaded from https://alphafold.ebi.ac.uk/ with corresponding Uniprot ID: G0RYB3. Annotation of the different domains of the model is shown. Color code represents pLDTT score as described in the Alphafold database. (B) PAE plot, showing high confidence for the structure prediction of all domains besides the flexible N- and C- termini. (C) Refined model in the density of the symmetry-expanded cryo-EM map. Model statistics are reported in Table S2. Fit shows frequent capturing of side chains, while other, often charged, side chains are missing due to high flexibility, overall positive map features due to the Coulomb potential map being calculated, and/or radiation damage.
